# PD88 Methods To Minimize Bias And Mediate Between Evidence And Preferences In Health Decision-Making: The Case Of Therapeutic Ranking

**DOI:** 10.1017/S0266462325102158

**Published:** 2025-12-29

**Authors:** Sandra Johanna Echeverry-Coral, Luis Esteban Orozco Ramírez, Giancarlo Romano Gómez, Sergio Rodrigo Basto Pacheco, Valeria Bejarano Salcedo, Nataly Arce Hernández, Mónica Nova Manosalva, Juan Camilo Vargas González, David Díaz-Báez

## Abstract

**Introduction:**

Therapeutic ranking (TR) is a process used in health technology assessment (HTA) by the Institute for Health Technology Assessment (IETS) in Colombia to optimize drug prescription through analyses of effectiveness, safety, costs, and post-marketing surveillance. However, decision-making relying on clinical expert preferences may introduce biases, since they are often influenced by subjective values. This study presented an alternative methodology by IETS that emphasizes a structured, evidence-based approach to improve decision-making in TR.

**Methods:**

The standard TR process involved forming an interdisciplinary group to assess the effectiveness, safety, costs, and post-marketing surveillance of medications, followed by evidence synthesis and informed voting using the Borda method to prioritize technologies based on consensus-driven preferences. To enhance informed voting, a methodological strategy was introduced that incorporated structured comparative tables and relative position scales derived from specific quantitative analyses for each parameter (e.g., surface under the cumulative ranking curve [SUCRA], costs, number of adverse events). This approach visually displayed performance patterns for integrated analysis by clinical experts.

**Results:**

This adaptation to TR consolidated the results for effectiveness, safety, costs, and economic evaluations of the assessed technologies. The structured format facilitated the identification of performance patterns by technology and enabled the integration of these results into a visual framework that supported deliberations during informed voting. This methodology promoted the integration of multiple evaluated dimensions, ensuring that inputs from epidemiology, pharmacology, health economics, and other specialized analyses were not overshadowed by the clinical experience of the expert panel. Instead, each input contributed to the collective decision-making process.

**Conclusions:**

This methodological approach enhanced TR as an HTA process by fostering evidence-based decisions. Optimizing the process could involve assigning weights to each analysis component, including clinical expertise, to balance their influence on prioritization. Explicit weighting would improve transparency and reproducibility. This model is replicable in other settings, maximizing HTA’s impact on global health systems.

